# *Mycoplasma cynos*‐Associated Canine Infectious Respiratory Disease Complex Pneumonia in 13 Dogs

**DOI:** 10.1111/vec.70042

**Published:** 2025-11-03

**Authors:** Danielle M. Williams

**Affiliations:** ^1^ VCA Highlands Ranch Animal Specialty and Emergency Center Highlands Ranch Colorado USA

**Keywords:** antimicrobial, dog, infectious disease, noncardiogenic

## Abstract

**Objective:**

To report the diagnostic results, treatments, and outcomes in 13 dogs with suspected canine infectious respiratory disease complex (CIRDC)‐associated pneumonia and *Mycoplasma cynos*–positive polymerase chain reaction (PCR).

**Design:**

Retrospective and prospective case series.

**Setting:**

Emergency and referral hospital.

**Animals:**

Thirteen client‐owned dogs with suspected CIRDC‐associated pneumonia and a positive PCR.

**Interventions:**

None.

**Measurements and Main Results:**

All dogs with history, clinical signs, and radiographs consistent with CIRDC‐associated pneumonia and a positive upper respiratory PCR that presented during a cluster from August 15, 2023, to September 9, 2023, were retrospectively and prospectively included in the series. Among the 13 dogs included, *M. cynos* was the sole pathogen detected in the airways of eight dogs, while *M. cynos* and *Bordetella bronchiseptica* were detected in five dogs. No viral etiologies were detected in this sample. Doxycycline, amoxicillin–clavulanic acid, and fluoroquinolones were frequently prescribed for ≥2 weeks. Follow‐up was performed until 1 week after resolution of cough, evidence of radiographic resolution, or death of the dog. Twelve dogs survived, and one was euthanized.

**Conclusions:**

*Mycoplasma cynos* was detected as the sole or co‐occurring infection during this 3‐week period, indicating it may be an important agent in some CIRDC clusters.

**Abbreviations**
CAV‐2: canine adenovirus type 2
CDV: canine distemper virus
CHV: canine herpes virus
CIRDC: canine infectious respiratory disease complex
CPIV: canine parainfluenza virus
CRCoV: canine respiratory coronavirus
PCR: polymerase chain reaction
TTWF: transtracheal wash fluid

## 1 Introduction

Canine infectious respiratory disease complex (CIRDC), or kennel cough, can be easily spread and can present with a complex etiology. Outbreaks occur when dogs are housed closely together in kennels, shelters, daycare facilities, or other high‐density dog locations. Some pathogens may be spread through fomites such as shared water bowls or toys [1]. Canine hosts may be infected with bacteria or viruses through saliva, urine, or aerosol transmission. Coinfections may also occur. Bacterial organisms associated with CIRDC include *Bordetella bronchiseptica*, *Streptococcus equi* subspecies *zooepidemicus*, and *Mycoplasma cynos*. Associated viral organisms include canine adenovirus type 2 (CAV‐2), canine parainfluenza virus (CPIV), canine influenza virus, canine herpes virus (CHV), canine distemper virus (CDV), canine pneumovirus, and canine respiratory coronavirus (CRCoV). Historically, *B. bronchiseptica* has been identified as the primary bacterial disease pathogen in CIRDC [2, 3, 4, 5, 6], while CPIV, CAV‐2, and CHV have been the main reported viral causes [4]. These agents, when coinfected with *M. cynos*, may cause more severe diseases in hosts [7].

*Mycoplasma* species are single‐cell commensal or opportunistic organisms that belong to the phylogenetic class Mollicutes [4]. They are the smallest free‐living organisms [8], lack a rigid cell wall, and can exist without oxygen in their environment or host; however, they may be difficult to culture [9]. Some *Mycoplasma* species are more hardy than others and may survive outside a host for more than 24 h because of their biofilm production [10]. Although *Mycoplasma* species can be difficult to culture, polymerase chain reaction (PCR) can be used to amplify their DNA, helping to identify infections [7, 11].

*Mycoplasma* species are present in the upper and lower respiratory tracts of healthy dogs and those with disease, making its role in CIRDC complicated [12]. *Mycoplasma bovigenitalium*, *M. canis*, *M. cynos*, *M. edwardii*, *M. feliminutum*, *M. gateae*, and *M. spumans* have all been detected in dogs with respiratory disease [13, 14, 15]. Of these, *M. canis*, *M. cynos*, *M. edwardii*, and *M. spumans* were isolated from the lower respiratory tract of dogs with CIRDC and were evaluated in a meta‐analysis by Waites et al. [8]. Of those isolates, only *M. cynos* was found to have a primarily pathogenic role. *Mycoplasma cynos* is thus sometimes a co‐pathogen in the lower respiratory tract of dogs [1, 6, 14, 16, 17]. A 2019 study revealed that 24.5% of CIRDC panels identified coinfections including *M. cynos* and that coinfections with CPIV were the most common, followed by *B. bronchiseptica* and CRCoV [18]. Upper respiratory airway sampling has also been studied, and while *Mycoplasma* species (e.g., *M. edwardii*) can be found in healthy dogs, *M. cynos* alone or in combination with other pathogens is commonly associated with severe respiratory disease in dogs [5, 17]. *Mycoplasma cynos* is also the only *Mycoplasma* species that can be cultured from the air [11], although it has yet to be determined whether dogs can be infected in vivo.

The aim of the current study was to report the diagnostic tests, treatments, and outcomes in 13 dogs with suspected CIRDC‐associated pneumonia and *M. cynos*–positive PCR that were presented during a short time period.

## 2 Materials and Methods

### 2.1 Case Selection and Review

Medical records from the emergency department of a referral specialty hospital were actively monitored beginning on August 15, 2023, during an uptick in cases of suspected CIRDC. Monitoring of records ended on September 9, 2023, when the caseload decreased. After three severe cases of suspected CIRDC‐associated pneumonia, including two deaths at the author's hospital, case monitoring was initiated to investigate the situation. Clinicians in the emergency department were asked to flag cases of suspected CIRDC‐associated pneumonia for use in the current report. Dogs were included if the attending clinician suspected CIRDC‐associated pneumonia based on a recent history of boarding, dog parks, grooming, or daycare, in addition to exhibiting consistent clinical signs (e.g., malaise, cough, fever, poor appetite), radiographic evidence of pneumonia, and positive PCR results. If radiographs did not reveal pneumonia in a suspected CIRDC case, if a PCR test was not performed, or if PCR results were negative, dogs were excluded from the study.

During the same time period the previous year (August 15, 2022, to September 9, 2022), no dogs presented to the hospital meeting the inclusion criteria of the current study, raising concern for a potentially more severe form of CIRDC in 2023. Written consent was obtained from the owners of the dogs for which essential identifying data were published in this report.

Follow‐up information was obtained through phone calls, email, and in‐person rechecks with dog owners, as well as phone call correspondence with the dogs’ primary care veterinarians and discussions of medical records with each dog's attending clinician. The dogs were followed up until they had clinical or radiographic evidence of resolution of disease, or upon death. No dogs were lost to follow‐up.

### 2.2 Samples

Decisions about diagnostic sampling were made by the attending clinician and the client. PCR samples were collected from sterile cultures using plastic‐stemmed swabs. For all dogs, the conjunctiva and the pharynx were swabbed, with care taken to avoid swabbing the oral mucosa. In five dogs, the nares were swabbed as well as the conjunctiva and pharynx. The swabs were then placed in sterile, red‐topped tubes without additives and stored in a refrigerator at approximately 4°C for transport to a commercial laboratory^1^ within 12 h of sampling. The tested agents included CDV, *B. bronchiseptica*, CAV‐2, CHV type 1, CPIV, CRCoV, H3N2 influenza virus, *M. cynos*, *S. equi* subspecies *zooepidemicus*, and canine pneumovirus.

Transtracheal aspiration was performed for transtracheal wash fluid (TTWF) sampling in two dogs in addition to conjunctival/nares/pharyngeal PCR testing. Transtracheal washing was performed routinely at the cricothyroid ligament using 1 mL/kg of sterile saline and a commercial transtracheal wash kit^2^. Samples were placed separately into sterile cultures and stored in a refrigerator at approximately 4°C for transport to a commercial laboratory^3^ within 12 h of sampling. Of the two TTFW samples, one was also submitted for *Mycoplasma* culturing at the Animal Health Diagnostic Center^4^.

## 3 Results

### 3.1 Signalment and Clinical Presentation

Dogs presented a median of 14 days after the onset of clinical signs (range: 7–56 days). The median age of dogs was 4 years (range: 7 months to 10 years), and the median weight was 25.5 kg (range: 13.6–38.2 kg). Breeds of the 13 dogs included five Labrador Retrievers, two Border Collies, two mixed breeds, one Australian Cattle Dog, one Golden Retriever, one Bloodhound, and one Australian Kelpie.

All dogs presented with an increased respiratory rate (>30/min) and cough, and 10 of 13 dogs developed fever >39.2°C (reference interval: 37.5°C–39.2°C). One dog was noted to have an increased temperature, but no value was recorded. Normal temperatures were measured in three dogs. Anorexia was noted in six dogs, five dogs were eating well, and two dogs’ eating habits on the day of presentation were unknown.

Four of 13 dogs were referred by a primary care veterinarian, and of those, four had received antimicrobial treatment (amoxicillin–clavulanic acid^5^) the week before presentation. Most dogs (seven of 13) had recently been boarded at various kennels, four dogs had been at various dog daycares, one dog had been at a dog park, and one dog recently had a grooming appointment. All dogs presented with their vaccines up to date, including PO, SC, or intranasal *B. bronchiseptica*. All dogs were previously healthy, although one had been diagnosed with CIRDC 3 years prior, and one had been diagnosed with CDV 8 years prior.

### 3.2 Diagnostic Results

All 13 dogs had CBC results available, and 12 dogs had biochemical results available; testing was done with in‐house analyzers^6^. The most common CBC abnormality was a leukocytosis (median: 14.7 × 10^9^/L [14,700/µL]; reference interval: 2.9–17.0 × 10^9^ [2900–17,000/µL]). The most common biochemical abnormality was hyperglobulinemia (median: 40 g/L [4.0 g/dL]; reference interval: 28–51 g/L [2.8–5.1 g/dL]). Twelve of 13 dogs had thoracic radiographs obtained on the day of presentation; radiographs were obtained the next day in the remaining dog. Variations in the radiologist's findings included pneumonia characterized by an alveolar pattern in all dogs, with eight of 13 having an alveolar pattern in the right middle lobe, six of 13 in the right cranial lobe, and six of 13 in the left cranial lobe. Two dogs had an alveolar pattern in the left caudal lobe only, and one dog had an alveolar pattern in the right caudal lobe only. There was an additional diffuse bronchointerstitial pattern noted in eight of 13 dogs.

Follow‐up thoracic radiographs were performed within 3 weeks of initial presentation in all but four dogs, for which the client declined consent or the dog was deceased. In all nine dogs, resolution of the alveolar pattern was seen on follow‐up thoracic radiographs. In addition, only three of the follow‐up radiographs showed a persistent bronchointerstitial pattern.

Thoracic point‐of‐care ultrasound was performed in two dogs. Scant free fluid was seen in one dog in the left hemithorax and was aspirated as serosanguineous fluid, although no further testing was performed. The other dog had multiple B‐lines that were more abundant on the left side than on the right.

Among the 15 dogs originally enrolled in the study, two were excluded because of negative PCR results. The upper respiratory samples in the 13 included dogs were 100% PCR positive for *M. cynos*. *Mycoplasma cynos* was the sole organism detected in eight of 13 dogs (61.5%), while *M. cynos* was detected along with *B. bronchiseptica* in five of 13 dogs (38.5%). No tested viruses were detected in any of the samples from this population of dogs. Four of the five dogs that tested positive for *B. bronchiseptica* had been vaccinated for *B. bronchiseptica* within the previous 6 months, and one dog had been vaccinated 7 months earlier.

Transtracheal sampling was performed in two dogs. In both dogs with transtracheal washes, the upper respiratory sample PCR tests were positive for both *M. cynos* and *B. bronchiseptica*. In one dog, TTWF was cultured aerobically and included add‐on sensitivities upon initial presentation. *Bordetella bronchiseptica* was the only organism isolated and showed resistance to amoxicillin–clavulanic acid, amoxicillin, ampicillin, cefadroxil, cefazolin, cefoxitin, cefpodoxime, ceftiofur, cephalexin, and cefovecin but showed susceptibility to enrofloxacin, amikacin, gentamicin, marbofloxacin, potentiated sulfonamides, azithromycin, and doxycycline. In the second dog, the TTWF was not sampled until recurrence of pneumonia, at which time the sample was cultured aerobically and for *Mycoplasma* species using *Mycoplasma*‐specific medium. *Pseudomonas aeruginosa* was the only isolated organism and was resistant to amoxicillin, ampicillin, cefadroxil, cefazolin, cefoxitin, cefpodoxime, ceftiofur, cephalexin, amoxicillin–clavulanic acid, cefovecin, and potentiated sulfonamides but was susceptible to amikacin, enrofloxacin, and marbofloxacin. Due to a delay in transport to the laboratories, determining whether *Mycoplasma* species were present in the TTWF was not possible because of overgrowth of other bacteria.

### 3.3 Treatments

Hospital admission was recommended in 11 of 13 cases and was accepted in seven. All hospitalized dogs received maropitant^7^ (1 mg/kg, IV, q 24 h) to improve appetite and prevent nausea, as well as antimicrobial treatment and IV fluid therapy. Four of the hospitalized dogs received doxycycline^8^ (5 mg/kg, IV, q 12 h) and ampicillin–sulbactam^9^ (30–50 mg/kg, IV, q 8 h). Enrofloxacin^10^ (10 mg/kg, IV, q 24 h) and doxycycline (5 mg/kg, IV, q 12 h) were prescribed for two dogs. Doxycycline alone (5 mg/kg, IV, q 12 h) was prescribed for one dog. Ampicillin–sulbactam alone (35 mg/kg, IV, q 8 h) was prescribed for one dog.

The four dogs whose owners declined hospitalization were treated on an outpatient basis with the following medications: maropitant (1 mg/kg, SC, once) in case the dog was nauseated, subcutaneous fluids, amoxicillin–clavulanic acid (13.75 mg/kg, PO, q 12 h) for 14 days, doxycycline (5–10 mg/kg, PO, q 12 h) for 14 days, and capromorelin^11^ (3 mg/kg, PO, q 24 h). A probiotic^12^ was prescribed for one dog. Outpatient therapy was recommended for two dogs by the attending clinician, with treatment including ampicillin–sulbactam (35 mg/kg, SC, once) in one dog. Both dogs received lactated Ringer's solution^13^ (SC), maropitant (1 mg/kg, SC, once), amoxicillin–clavulanic acid (13.75 mg/kg, PO, q 12 h) for 14 days, doxycycline (5 mg/kg, PO, q 12 h) for 14 days, and capromorelin (3 mg/kg, PO, q 24 h).

### 3.4 Clinical Outcomes

Twelve of 13 dogs had resolution of their pneumonia and survived. Resolution of cough occurred after a median of 9 days (range: 3–73 days), and radiographic resolution was seen after a median of 11 days (range: 8–15 days). One dog received antimicrobials for 2 weeks, seven of 13 dogs for 3 weeks, one dog for 4 weeks, and one dog for 6 weeks. A short course of prednisone was given to one dog for a persistent cough, leading to resolution.

Among the 13 dogs, an 8‐year‐old neutered male Australian Cattle Dog was euthanized because of a grave prognosis. The dog, previously healthy but diagnosed with CDV 8 years prior, presented to the emergency room after boarding for 2 weeks. The dog had been coughing for 3 days and was tachypneic on presentation without increased effort. The dog's temperature was 38.1°C. The dog was treated as an outpatient, and blood test results were unremarkable with the exception of bands and slight lymphopenia, eosinopenia, and hypochloremia. The owner declined thoracic radiographs on the day of presentation but approved PCR testing of canine upper respiratory samples, which revealed *M. cynos* as the sole organism detected. The dog was discharged with amoxicillin–clavulanic acid and doxycycline; however, the family was unable to administer the medications. Within 24 h, the dog was referred to another hospital due to progressive tachypnea and lethargy. Thoracic radiographs were performed, which revealed a severe alveolar pattern that encompassed ∼80% of the pulmonary parenchyma. Results of blood tests suggested sepsis, characterized by neutropenia, hyperlactatemia, and hypoglycemia. The dog rapidly declined, and humane euthanasia was recommended by the attending clinician. Necropsy was not performed on this dog.

## 4 Discussion

This study describes 13 dogs that presented within a short time period, all suspected of having CIRDC‐associated pneumonia and all of which had PCR‐detected *M. cynos*. The rate at which *M. cynos* alone was detected in this report is higher (61.5%) than previous studies of CIRDC‐associated pneumonia (32%, 24.5%, and 49.5%) [16, 18, 19]. The current study provides evidence that *M. cynos* may be a primary agent in some disease clusters. This study also suggests that in dogs with suspected CIRDC‐associated pneumonia, upper respiratory canine PCR may be a useful diagnostic test for *M. cynos* and *B. bronchiseptica*, though its limitations should be considered. *Mycoplasma cynos* is more frequently isolated in lower respiratory tract samples from sick dogs [9]; therefore, bronchial or tracheal lavage and culture may be superior to upper respiratory swabs for its detection. However, PCR testing of upper respiratory samples was chosen by these clients given the relatively low cost, as well as the test's minimally invasive nature. Of the TTWF cultures evaluated, one grew *B. bronchiseptica* but not *Mycoplasma* species, despite a positive *M. cynos* PCR result for upper respiratory samples. *Mycoplasma* species are fragile; thus, PCR testing is preferred over culture for this organism [8]. A study in people by Reizenstein et al. found that sensitivity for *B. bronchiseptica* was also lower in cultures than on PCR testing [20]. The other TTWF sample was submitted for culture after 3 weeks of antimicrobials and pneumonia recurrence, and revealed *P. aeruginosa*, a highly resistant gram‐negative opportunistic organism that was susceptible only to amikacin, enrofloxacin, and marbofloxacin. *Pseudomonas aeruginosa* is designated by the World Health Organization as a priority organism because of its high levels of antimicrobial resistance [21]. It is typically found in the environment and can colonize hosts that have a breach in the body's defenses, such as immunocompromised dogs. Both *B. bronchiseptica* and *M. cynos* were originally detected in this dog. We now know that some *Mycoplasma* species, including *M. cynos*, have sialidase activity [22], which has a direct toxic effect on host cells and defense mechanisms [23]. *Pseudomonas aeruginosa* is suspected to have colonized the dog's injured lungs secondary to the original infection.

This report adds to the current literature by revealing that *M. cynos* is a common pathogenic organism in the etiology of CIRDC, perhaps even more so than viruses and *B. bronchiseptica* in certain outbreaks. CIRDC can affect dogs of any age and breed; however, young, large breed dogs appeared overrepresented in the current study and were more severely affected than in previously described clusters. Labrador Retrievers accounted for five of 13 breeds in this report; the high number of larger‐breed dogs may have been related to environmental factors—for instance, possibly more large breed dogs visit dog parks in our area. All dogs were vaccinated according to current American Animal Hospital Association guidelines, and this may have decreased the viral etiology. It should also be noted that PCR tests may not capture the viral shedding window.

Pneumonia resulting from *Mycoplasma* species has been previously documented [7, 19, 24, 25]. Canine pneumonia has been experimentally reproduced by the endobronchial administration of *M. cynos* [15], but it has not yet been proven to be a naturally occurring primary disease pathogen in CIRDC‐associated pneumonia. Pulmonary thromboembolism and aspiration are among the known predisposing causes for pneumonia, potentially by reducing the bactericidal activity of neutrophils [19]. Although there were some historical diseases in this population, there was no direct evidence that they affected the outcomes.

The results of the current study suggest that upper respiratory PCR sampling should be considered to help detect potential CIRDC etiologies, especially because common antimicrobials such as amoxicillin–clavulanic acid do not affect *M. cynos*. Considering the organisms present, culturing tracheal or bronchial samples may be beneficial in determining the ideal antimicrobial choice, although *M. cynos* is fastidious and may be difficult to culture. The mechanism of action of penicillin includes the interruption of penicillin‐binding proteins in the cell wall of the infecting organism. *Mycoplasma* species lack a cell wall; therefore, they are inherently resistant. In addition, the species are susceptible to macrolides, azalides, tetracyclines, chloramphenicol, lincosamides, and fluoroquinolones [26]. Amoxicillin–clavulanic acid has a mechanism of action to which *B. bronchiseptica* should be susceptible, but resistance was seen in the TTWF of one of the dogs in the current report [27].

Based on the inherent resistance of *Mycoplasma* species and clinical resistance of *B. bronchiseptica* to amoxicillin–clavulanic acid, doxycycline is recommended by the International Society for Companion Animal Infectious Diseases respiratory guidelines as the first‐line treatment [28]. Until further evidence is obtained, the author recommends using these guidelines as a basis for the treatment of CIRDC; however, the antimicrobials prescribed for the dogs in the current study did not always adhere to these guidelines.

There is conflicting evidence regarding the optimal duration of treatment for canine pneumonia. Some studies on uncomplicated pneumonias provide evidence that there is no outcome difference when comparing short‐ and long‐course antimicrobials [29, 30]. One study recommends a 10‐day course of therapy for uncomplicated pneumonia and suggests that evidence of radiographic resolution is misleading [31]. However, other studies recommend treatment be continued for 1–2 weeks beyond evidence of radiographic resolution [32, 33]. *Mycoplasma cynos* can persist in the lung for up to 3 weeks after infection [10], and some *Mycoplasma* species form a biofilm that may allow them to persist even longer [34]. This may explain why some *Mycoplasma* species cannot be completely removed from the host, even with the use of antimicrobials to which the organisms are susceptible. Given what is known of *M. cynos*, and that 8 of 13 dogs successfully treated in this study were administered antimicrobials for more than 2 weeks, the author recommends further investigation into the duration of antimicrobial use in dogs with CIRDC. C‐reactive protein testing may also be added to help determine the appropriate timing for the withdrawal of antimicrobials. A study of dogs with aspiration pneumonia revealed that this type of pneumonia can also be treated with short‐course antimicrobials, that C‐reactive protein testing should be considered, and that clinical improvement should be considered when deciding the length of antimicrobial treatment [35].

The current series has some limitations. The animals in this series represent a subpopulation of dogs with suspected CIRDC‐associated pneumonia identified in a specific region and evaluated at one referral hospital; this inherent bias prevents broad application of the findings to the overall population of dogs with CIRDC and general practices. Although all the dogs in this report presented with a cough, it is a nonspecific sign, and comorbidities were not always ruled out. Using PCR to test upper respiratory samples may not reveal agents within the lower airways, even potential causal, consequential, and independent carriers of *M. cynos*. Depending on the organism, dogs may be tested after the shedding period. Another limitation was the small sample size and the inability to culture each sample or to perform PCR testing of upper respiratory samples, due to financial constraints. No cytologic samples were evaluated from the lower airways; other bacterial species could have been present but were therefore undetected. A more robust sample size may yield a different trend in the findings. However, in this cluster of dogs with CIRDC, *M. cynos* was detected more frequently and was associated with more severe disease than previously reported. No viruses specifically tested for in this study were found, and *M. cynos* was more common than *B. bronchiseptica*.

Current guidelines for the treatment of respiratory diseases in dogs state that most cases of CIRDC are believed to be viral in etiology, and antimicrobial administration is often not indicated [28]. Given the findings in this CIRDC cluster, clinicians should use medications based on findings from local PCR results and based on International Society for Companion Animal Infectious Diseases recommendations. The development of resources to conduct surveillance for community‐based disease outbreaks in veterinary medicine, whether at the private practice, state, or national levels, is encouraged. The results of this study offer insights into the strategies for the diagnosis and treatment of CIRDC. Further research is needed to test for novel viruses and organisms that may be present in CIRDC that are not accounted for by currently available diagnostic tools.

## Author Contributions

**Danielle M. Williams**: conceptualization, data curation, investigation, methodology, project administration, resources, software, supervision, validation, visualization, writing – original draft, writing – review and editing.

## Ethics Statement

The author confirms that the ethical policies of the journal, as noted on the journal's author guidelines page, have been adhered to. No ethical approval was required given the nature of this report, which demonstrates a high standard (best practice) of veterinary care and involves informed client consent.

## Conflicts of Interest

The author declares no conflicts of interest.

## Endnotes

1 IDEXX Laboratories, Inc., Westbrook, ME.

2 Mila International Inc., New York, NY.

3 ANTECH Diagnostics, Inc., Sheridan, CO.

4 Cornell University College of Veterinary Medicine, Ithaca, NY.

5 Amoxicillin–clavulanic acid, Zoetis Inc., Kalamazoo, MI.

6 ProCyte Hematology Analyzer, Catalyst One Veterinary Blood Chemistry Analyzer; Idexx Laboratories, Inc., Westbrook, ME.

7 Maropitant, Zoetis Inc., Kalamazoo, MI.

8 Vibramycin, Pfizer Inc., New York, NY.

9 Ampicillin–sulbactam, Pfizer Inc., New York, NY.

10 Enrofloxacin, Elanco Inc., Greenfield, IN.

11 Capromorelin, Elanco Inc., Greenfield, IN.

12 Visbiome, Exegi Pharma, LLC., Rockville, MD.

13 Lactated Ringer's Solution, ICU Medical Inc., San Clemente, CA.
